# Sustained Epigenetic Reactivation in Fragile X Neurons with an RNA-Binding Small Molecule

**DOI:** 10.3390/genes16030278

**Published:** 2025-02-25

**Authors:** Christina W. Kam, Jason G. Dumelie, Gabriele Ciceri, Wang-Yong Yang, Matthew D. Disney, Lorenz Studer, Samie R. Jaffrey

**Affiliations:** 1 Department of Pharmacology, Weill Medical College, Cornell University, New York, NY 10065, USA; 2 The Center for Stem Cell Biology, Sloan Kettering Institute for Cancer Research, New York, NY 10065, USA; 3 Developmental Biology Program, Sloan Kettering Institute for Cancer Research, New York, NY 10065, USA; 4 Department of Chemistry and Physics, University of Tennessee at Chattanooga, Chattanooga, TN 37403, USA; 5 Department of Chemistry, The Scripps Research Institute, Scripps Florida, Jupiter, FL 33458, USA; 6 Department of Chemistry, The Herbert Wertheim UF Scripps Institute for Biomedical Innovation & Technology, Jupiter, FL 33458, USA

**Keywords:** fragile X syndrome, RNA-binding small molecule, epigenetics

## Abstract

Background/Objectives: Fragile X syndrome (FXS) is a disease of pathologic epigenetic silencing induced by RNA. In FXS, an expanded CGG repeat tract in the *FMR1* gene induces epigenetic silencing during embryogenesis. *FMR1* silencing can be reversed with 5-aza-deoxyctidine (5-aza-dC), a nonspecific epigenetic reactivator; however, continuous administration of 5-aza-dC is problematic due to its toxicity. We describe an approach to restore *FMR1* expression in FXS neurons by transient treatment with 5-aza-dC, followed by treatment with 2HE-5NMe, which binds the CGG repeat expansion in the *FMR1* mRNA and could block the resilencing of the *FMR1* gene after withdrawal of 5-aza-dC. Methods: This study uses immunofluorescence and fluorescent in situ hybridization (FISH) to measure whether *FMR1* expression is maintained in FXS post-mitotic neurons treated with 2HE-5NMe. Genome-wide profiling of histone marks was used to monitor epigenetic changes and drug selectivity in response to 5-aza-dC followed by 2HE-5NMe treatment. Changes to dendritic morphology were visualized using confocal microscopy. Results: In this study, we find that 2HE-5Nme maintains *FMR1* in a reactivated state after reactivation using 5-aza-dC in post-mitotic neurons. *FMR1* reactivation in neurons results in the re-expression of FMRP and reversal of FXS-associated dendritic spine defects. Conclusions: These results demonstrate that an RNA-binding small molecule can achieve gene-specific epigenetic control and provide an approach for the restoration of FMRP in FXS neurons.

## 1. Introduction

Abnormal epigenetic silencing contributes to gene expression abnormalities in diverse diseases. This is particularly prominent in fragile X syndrome (FXS), which is caused by an expansion of a CGG repeat tract in the *FMR1* gene, which was discovered by Warren and colleagues [1,2]. The CGG repeat expansion is located in the region of the gene corresponding to the 5′UTR and results in the epigenetic silencing of the *FMR1* gene [3]. Epigenetic silencing occurs at ~11 weeks of gestation, which results in the termination of *FMR1* mRNA transcription and a subsequent loss of the encoded protein, FMRP [4]. The lack of FMRP results in immature thin dendritic spines in neurons, which is a classic feature of FXS [5]. A major therapeutic goal is to selectively reverse the epigenetic silencing of *FMR1* in FXS and thus restore FMRP expression and potentially reverse the abnormal neuronal morphology seen in FXS.

RNA is required for the epigenetic silencing of *FMR1*. The role of the *FMR1* transcript in mediating *FMR1* gene silencing was first discovered during studies of FXS human embryonic stem cells (hESCs) [6]. FXS hESCs mimic the early embryonic state of FXS in which the *FMR1* locus is active despite the presence of >200 CGG repeats. However, during the differentiation of FXS hESCs, the *FMR1* locus undergoes epigenetic silencing, resulting in a loss of FMRP protein. The CGG repeat portion of the *FMR1* mRNA interacts with the complementary repeat region in the DNA to form an RNA–DNA hybrid, also known as an R-loop, which subsequently triggers gene silencing. Notably, a CGG repeat RNA-binding small molecule can disrupt R-loop formation and prevent the epigenetic silencing of the *FMR1* gene in hESCs [6]. These studies have established the importance of the CGG repeat RNA in *FMR1* silencing.

Currently, the only pharmacologic agent that substantially reactivates *FMR1* in FXS is 5-aza-dC, a cytosine analog that non-selectively induces gene expression by interfering with the pathways that lead to epigenetic gene silencing [7]. This finding has been confirmed with more recent drug screens, which show that 5-aza-dC is one of the most robust reactivators of *FMR1* expression relative to other small molecule epigenetic regulators [8,9]. 5-aza-dC and related cytosine analogs partially reactivate the expression of *FMR1* in FXS lymphoblastoid cells, fibroblasts and neural progenitor cells [10,11,12,13].

5-aza-dC is incorporated into DNA during mitosis, which then traps and inactivates DNA methyltransferases that attempt to use the DNA-incorporated 5-aza-dC as a substrate for methylation [14]. As a result, epigenetic silencing cannot be established on newly synthesized DNA, resulting in inefficient silencing after mitosis. Notably, a major cell type affected in FXS is neurons, which do not undergo mitosis. However, 5-aza-dC has previously been shown to modulate the expression of *BRCA1*, *reelin, BDNF* and other genes in post-mitotic neurons, through an unknown mechanism [15,16,17]. It is therefore unclear whether 5-aza-dC can remove epigenetic silencing in the context of the *FMR1* CGG repeat expansion in post-mitotic cells.

Here, we describe a strategy to selectively reactivate the silenced *FMR1* allele in FXS neurons and induce FMRP synthesis. In this approach, we use 5-aza-dC, which we unexpectedly find restores *FMR1* expression in post-mitotic FXS neurons. Notably, the reactivation of *FMR1* expression leads to the restoration of FMRP protein expression. *FMR1* expression is maintained only as long as neurons are treated with 5-aza-dC, and silencing is restored when 5-aza-dC is withdrawn. Since continuous treatment with 5-aza-dC is associated with cytotoxicity, we developed an approach to maintain *FMR1* reactivation elicited by brief treatment with 5-aza-dC. We show that 2HE-5Nme, a CGG repeat-binding small molecule [18], maintains *FMR1* mRNA expression even after the withdrawal of 5-aza-dC. Genome-wide analysis of H3K4me3 and H3K27me3 marks show that 2HE-5Nme selectively maintains epigenetic activation of *FMR1*. 2HE-5Nme additionally maintained FMRP protein expression to FXS neurons, resulting in the normalization of dendritic spine morphology. Overall, these data demonstrate selective epigenetic reactivation of *FMR1* using an RNA-binding small molecule in FXS.

## 2. Methods

### 2.1. Quantitative Real-Time Polymerase Chain Reaction

Cells were lysed in TRIzol (Invitrogen, Waltham, MA, USA), and RNA was extracted following the manufacturer’s instructions. The RNA was further purified using an RNAeasy (Qiagen, Germantown, MD, USA) column, in the presence of DNaseI (RNase-free DNaseI, Qiagen). A Superscript III First-Strand Synthesis Kit (Invitrogen) with oligo(dT) was utilized to produce cDNA. Quantitative RT-PCR reactions were run in triplicate to evaluate 20 ng of cDNA utilizing iQ SYBR Green Supermix (BioRad, Hercules, CA, USA) on an Eppendorf Mastercycler ep realplex thermocycler. The primers that were used to quantify FMR1 mRNA and housekeeping gene GAPDH mRNA were as follows: FMR1 F: 5′GTATGGTACCATTTGTTTTTGTG 3′, FMR1 R: 5′ CATCATCAGTCACATAGCTTTTTTC 3′; GAPDH F: 5′ AGCCACATCGCTCAGACACC 3′ and GAPDH R: 5′ GTACTCAGCGGCCAGCATCG 3′.

### 2.2. Western Blot

The fibroblast dishes were incubated on ice and washed with ice-cold PBS supplemented with 1× protease inhibitors (Roche, Basel, Switzerland) and 1× phosphatase inhibitors (Sigma, St. Louis, MO, USA). Adherent cells were removed with a cold cell scraper and transferred to a pre-cooled microcentrifuge tube on ice. The fibroblasts were then lysed in RIPA buffer (50 mM Tris [pH 8.0], 150 mM NaCl, 1% Nonidet P-40, 0.1% SDS) supplemented with 1× protease and 1× phosphatase inhibitors for 30 min at 4 °C. Centrifugation was performed for 10 min at 4 °C, and the supernatant was isolated. Total protein in the supernatant was quantified with the Pierce BCA Protein Assay kit (Thermo, Waltham, MA, USA). A total of 10 or 20 micrograms of total protein was loaded in each lane of a NuPAGE Bis-Tris 4–12% Gel (Invitrogen). Proteins were resolved on the gel and transferred to the PVDF membrane. Membranes were blocked in 5% (wt/vol) nonfat milk and probed with the appropriate primary (FMRP–ab17722; GAPDH–ab9485) and secondary antibodies with washes in between. Pierce ECL Western blotting Substrate (Thermo) was utilized to produce chemiluminescence, and the membrane was imaged in a BioRad GelDoc.

### 2.3. ViewRNA Cell Plus

The ViewRNA Cell Plus (Thermo) kit was used following the manufacturer’s instructions. Samples were plated in 35 mm Mattek glass-bottom dishes. Following the fixation and permeabilization of cells with the kit’s buffers, immunofluorescence was performed with antibodies to FMRP (MAB2160, Millipore, Burlington, MA, USA) and actin (sc-47778, Santa Cruz Biotechnology, Santa Cruz, CA, USA) or MAP2 (AB5622, Millipore). Type 6 FMR1 ViewRNA probes conjugated to Alexa 647 were hybridized, and then the signal was amplified following the manufacturer’s instructions. DAPI was added. Mounting was performed with ProLong Diamond Antifade Mountant (Invitrogen) by putting a circular glass coverslip over the glass bottom of the dishes. Dishes were immediately visualized by confocal microscopy.

### 2.4. Cell Culture and Neuronal Differentiation

Control (MRC5) and FXS lung fibroblast lines (GM07072) were cultured in DMEM supplemented with DMEM with 15% fetal bovine serum and penicillin/streptomycin.

Control (GM06890) and FXS (GM06852) lymphoblasts were cultured in RPMI with 20% fetal bovine serum and penicillin/streptomycin.

Control (WCMC-7) and FXS human embryonic stem cell lines (WCMC-37 and SI-214) were used with the approval of and in accordance with the ethical regulations of the Weill Cornell Medicine Embryonic Stem Cell Research Oversight (ESCRO) Committee. hESCs were cultured on an MEF layer in hESC medium (DMEM/F12 [Invitrogen], 20% knockout serum replacement [Invitrogen], 0.1 mM 2-mercaptothanol, 1× MEM non-essential amino acids [Gibco, Waltham, MA, USA], 200 mM L-Glutamine) supplemented with 10 ng/mL FGF-2 [StemBeads, StemCulture].

Human embryonic stem cells were dissociated into single cells using Accutase (Stem Cell Tech) and plated at high density on Matrigel (Corning, Corning, NY, USA) in hESC medium supplemented with Y-27632 to improve cell survival. Cells were cultured for 10 days in the presence of LDN193189 (#72142, Stem Cell Technologies, Vancouver, BC, Canada), SB431542 (#1614, Tocris, Minneapolis, MN, USA) and XAV939 (#3748, Tocris) (until day 4). Afterward, cells were maintained in N2-supplemented DMEM/F12 until day 20. Neurons were replated following dissociation with Accutase onto PLO/laminin/fibronectin-coated plates in neuronal media (Neurobasal media, B27–vitamin A, Glutamax) supplemented with DAPT, BDNF, GDNF, ascorbic acid and cAMP, as previously described [16]. Neuronal media were replaced every 4–5 days. The confluency of plating depended on the intended use of cells (for imaging 65 K/cm^2^ and for CUT&RUN 150 K/cm^2^).

### 2.5. CUT&RUN

CUT&RUN was performed following the published protocol. On day 21 of neuronal differentiation, neurons were plated at 150 K/cm^2^. A total of 300,000 FXS neurons were harvested, washed and bound to beads. Neuron beads were divided into three aliquots for determining the binding locations of IgG control, H3K4me3 and H3K27me3. Neurons were permeabilized, and the appropriate primary antibodies were utilized. Protein A-MNase fusion protein (a gift of the Henikoff lab) was then added to bind to primary antibody locations. Targeted digestion was activated with addition of calcium and occurred for 5 min. Cut fragments were then released from insoluble nuclear chromatin and extracted using NucleoSpin columns (Takara, San Jose, CA, USA) following the manufacturer’s instructions.

Size selection was performed using AMPure XP beads (Agencourt, Beverly, MA, USA) before library preparation with the Kapa Hyper Prep Kit (Roche) following the manufacturer’s instructions. End repair and A-tailing were performed before adapter ligation with TruSeq (Illumina, San Diego, CA, USA) indices. The libraries were amplified, and then quality control was performed to ensure that primer dimers were negligible. Afterward, 150-base pair-end sequencing was performed with HiSeq by the Genomics Core Facility of the WCM Core Laboratories Center.

We used an alignment strategy that eliminates reads that align to any mouse transcripts, since some mouse feeder cells may have remained in the sample used for CUT&RUN. Paired-end reads were aligned to human genome version GRCh38.p13 using Bowtie2. Reads were normalized for sizes of libraries. Reads were then mapped onto a list of all protein-coding gene promoters (−1000 to +1000 of MANE Select transcription start sites from GRCh38.p13).

### 2.6. RNA-seq

WCM37 or SI-214 cells were plated on 35 mm dishes and differentiated as described above. The differentiated neurons were next incubated in 5-aza-dC (1 µM) for 7 days. Media was then used to wash out 5-aza-dC, and then the neurons were incubated in media with 2HE-5NMe or vehicle (DMSO) for 14 days. Media was removed from cells, and then RNA was extracted from the cells using Trizol (Invitrogen, 15596026) following the manufacturer’s instructions. The RNA was precipitated using isopropanol, washed with 70% ethanol and resuspended in water. The RNA (100 ng) was then converted into a cDNA library using the SMART-seq mRNA LP (with UMIs) kit (Takara, 634762) following the manufacturer’s instructions. The quality of the library was assessed using a High-Sensitivity D5000 ScreenTape assay (Agilent, Santa Clara, CA, USA), and then the cDNAs were sequenced using a NovaSeq 6000 with S1 Flow Cells (paired end, 2 × 50 bp).

In order to remove reads that originate from mouse feeder cells, sequencing reads were first aligned to the mouse genome (GRCm38) using hisat2 (2.2.1) with default settings. Reads that did not align to the mouse genome were then aligned to the human genome (GRCh38). The number of reads that aligned to each gene were calculated using HTSeq with -a set to 0 and all other settings set to default. Genes with low median expression (<10 reads per million) were removed from the analysis. To identify genes that might have significant gene expression changes between vehicle and 2H treatment, we used DESeq2 (1.34.0). Correlation and volcano plots were generated using ggplot2(3.3.5) in R (4.1.2).

### 2.7. Dendritic Spine Imaging

Neurons were plated at 65 K/cm^2^ on glass-bottom 12-well plates on day 21 of neuronal differentiation. Differentiation was continued until day 40. Starting on day 40, drug treatment (or DMSO vehicle) was administered while media was changed regularly. Two days before imaging, Lipofectamine 2000 (Life Tech, Carlsbad, CA, USA) was used to transfect GFP.

On the day of imaging, media was removed, and transfected neurons were fixed in 4% paraformaldehyde for 10 min at room temperature. Three washes were performed with 1× PBS for 10 min each. Neurons were permeabilized and blocked simultaneously in PBS containing 2% normal goat serum and 0.1% Triton-X-100 for 1 h at room temperature. Neurons were incubated with a 1:1000 dilution of a primary GFP antibody (Abcam, Cambridge, UK, ab13970–100 uL) overnight at 4 °C. Washes were performed 3×. Neurons were then incubated with a 1:200 dilution of an Alexa 488-conjugated secondary goat anti-chicken (Thermo, A11039) antibody for an hour at room temperature. Mounting was performed with ProLong Diamond Antifade Mountant (Invitrogen) by putting a circular glass coverslip over the glass bottoms of the dishes. Dishes were immediately visualized by confocal microscopy.

### 2.8. BrdU Immunofluorescence

WCM37 or SI-214 cells were plated on 35 mm dishes and differentiated as described above. The differentiated neurons were next incubated in BrdU (10 µM, Abcam, ab142567) ± 5-aza-dC (1 µM) for 48 hr. Media was then removed from the cells, and cells were washed in PBS (4×, 800 µL). The cells were fixed in formaldehyde (4%, 10 min) and then washed in PBS (3×, 800 µL). Next, cells were treated with 0.2% triton in PBS (10 min) and then washed in PBS (3×, 800 µL). The cells were then incubated in HCl (2N, 30 min) and washed in PBS (3×). The cells were then blocked for 10 min (2% FBS in PBS). Next, the cells were incubated (overnight, 4 °C) with 1:250 rat anti-BrdU (Abcam, ab6326) and 1:1000 rabbit anti-MAP2 (EMD Millipore, ab5622) in PBS with 2% FBS. Next, the neurons were washed in PBS (3×) and then incubated in goat anti-rabbit 647 (1 in 1000, A21245) and donkey anti-rat 488 (1 in 1000, A21208). Cells were washed in PBS (3×) and then incubated in DAPI (10 min). Finally, cells were washed, and coverslips were added to the cells.

### 2.9. Microscopy

Fluorescence microscopy to clarify neuronal identity was performed with a wide-field fluorescent microscope (Eclipse TE2000-E microscope, Nikon, Tokyo, Japan). Images were analyzed with NIS-Elements Viewer software v. 5.21.00 (Nikon).

ViewRNA and dendritic spine imaging experiments were performed using an LSM 880 laser scanning confocal microscope (Zeiss, Oberkochen, Germany) with Airyscan high-resolution detector. Z-stacks were taken from the top to the bottom of individual neurons at 63× oil immersion objective. Analysis was performed with ZEN Black software 2.3 (Zeiss) and Fiji (ImageJ v1.51n).

Three-dimensional visualization of example images of wild-type and FXS dendritic spines was performed with Imaris and Blender software 10.2.

BrdU microscopy was performed using the Inverted LSM 880 Airyscan NLO laser scanning confocal and multiphoton microscope (Zeiss) at the Bio-Imaging Resource Center at Rockefeller University. For each replicate, five to ten Z-stacks were imaged with exposure times that were below pixel saturation. The location of Z-stacks was determined exclusively by examining the sample using the MAP2-Alexa 647 Fluor-associated channel, to prevent unintentional bias in assessing whether MAP2-associated neurons were BrdU-labeled. Z-stacks were imaged using a 63× lens with Immersion Oil.

BrdU images were processed with the FIJI (2.3.0) distribution of ImageJ2 (1.53q). Deconvolution was performed on the images using the ImageJ DeconvolutionLab2 package (2.1.2) with ten iterations of the Richardson–Lucy algorithm. The point spread function was calculated using the PSF Generator (1.1.1.2) based on the Born and Wolf 3D optical model. The parameters to generate each point spread function matched the experimental condition, and accuracy computation was set to “Best”.

## 3. Results

### 3.1. 5-aza-dC Restores FMRP Expression in Post-Mitotic Neurons

We first confirmed that 5-aza-dC can reactivate *FMR1* mRNA expression in dividing FXS cells. The treatment of FXS fibroblasts for 7 days with increasing concentrations of 5-aza-dC, ranging from 0.25 µM to 5 µM, showed maximal reactivation of *FMR1* mRNA levels at 1 µM 5-aza-dC (Figure S1A). This effect requires the continuous presence of 5-aza-dC as *FMR1* expression was lost within 14 days after replacement with media lacking 5-aza-dC (Figure S1B). These data are consistent with earlier 5-aza-dC reactivation studies [10,11,12] and indicate that *FMR1* reactivation persists only if 5-aza-dC is maintained in the culture media.

Despite the 5-aza-dC-induced increase in *FMR1* transcript expression to wild-type levels, no FMRP protein was detected (Figure S1C), as reported previously [11]. This may reflect the inability of the ribosome to scan through the CGG repeat tract in the 5′UTR to the start codon. Overall, these experiments indicate that 5-aza-dC may have limited utility for *FMR1* reactivation due to its requirement for its continuous presence and the lack of FMRP protein expression, at least in fibroblasts.

5-aza-dC can affect gene expression through replication-independent mechanisms, including proteasomal degradation of DNA methyltransferases and associated epigenetic regulators [19]. We therefore asked if 5-aza-dC could induce *FMR1* reactivation in neurons derived from FXS hESCs. We prepared FXS neurons by differentiating FXS and wild-type hESCs [6] using a neuronal differentiation protocol. This protocol is based on dual-SMAD inhibition [20] in combination with Wnt inhibition, which is followed by Notch inhibition. It efficiently generates post-mitotic cortical neurons by day 30 of differentiation (Figure S1D) [21]. We previously used a 45-day approach involving dual-SMAD inhibition only (Noggin/TGFß inhibition) to differentiate FXS hESCs to neurons, which was used to show that the differentiation of FXS hESCs leads to *FMR1* silencing and the loss of detectable FMRP protein [6]. In the current study, we instead used the modified SMAD/Wnt inhibition protocol due to the higher fraction of cells that are differentiated to neurons and the more rapid differentiation into post-mitotic and functional cortical neurons [21,22,23]. Importantly, these differentiation conditions produce neurons with mature electrophysiological properties [23]. We confirmed that the neurons generated by the SMAD/Wnt inhibition protocol similarly showed an absence of *FMR1* mRNA and FMRP protein expression (Figure S1E).

Although neurons are known to be post-mitotic, we confirmed that the cells were indeed neurons and had differentiated to the post-mitotic stage. After forty days of maturation, we found that the neurons exhibited MAP2 staining throughout the soma and dendrites, verifying that they were post-mitotic neurons (Figure S1E) [24]. As expected, other cells in culture were not labeled with these markers, which reflects feeder cells or hESC-derived cells that differentiated to non-neuronal cells (Figure S1E) [25,26]. We therefore focused our analysis on cells that showed clear neuronal morphology and expressed MAP2. Overall, these experiments indicate that the differentiation protocol yields neurons that are fully differentiated and post-mitotic.

**Figure 1:**
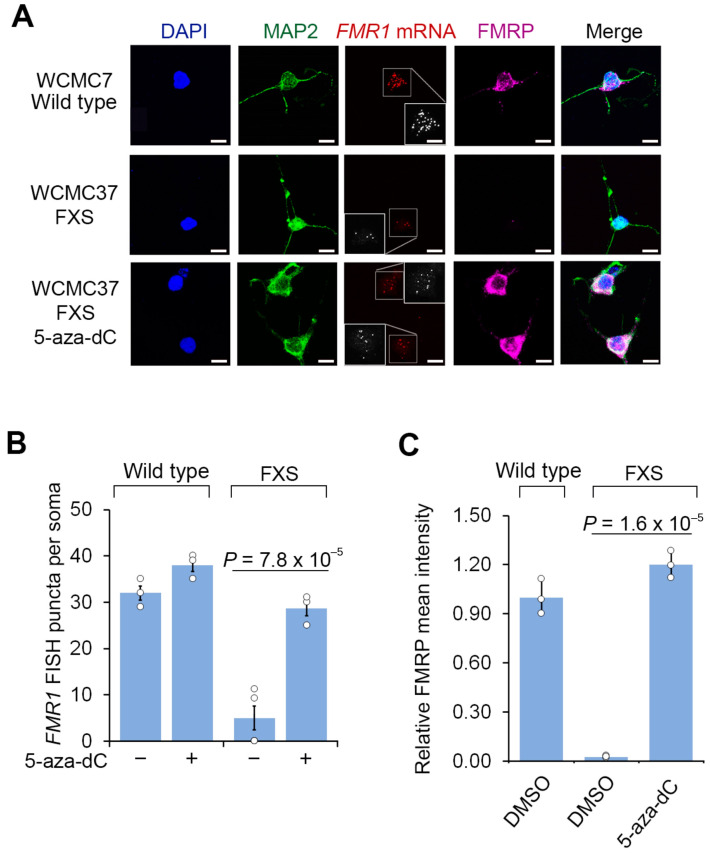
5-aza-dC reactivates *FMR1* mRNA expression and FMRP protein levels in FXS neurons. (**A**) FXS neurons derived from WCMC37 hESCs lack *FMR1* mRNA expression and FMRP, and 5-aza-dC reactivates expression. Representative images (*n* = 3 experiments) of anti-MAP2 and anti-FMRP immunofluorescence staining and *FMR1* FISH imaging of untreated wild-type and FXS neurons and FXS neurons treated with 1 µM 5-aza-dC for 7 days (scale bar, 10 µm). Insets show images enlarged by 150% with FISH puncta in white. (**B**) 5-aza-dC reactivates *FMR1* mRNA expression in FXS neurons. *FMR1* FISH was performed in neurons derived from a wild-type hESC line (WCMC7) and an FXS hESC line (WCMC37). 5-aza-dC (7 days, 1 µM) increases the number of detected *FMR1* FISH puncta in FXS neurons, but it has minimal effect on wild-type neurons. *FMR1* FISH foci were quantified from *n* = 3 experiments. Shown are the mean and s.d.; univariate two-sided *t*-test. (**C**) 5-aza-dC treatment leads to the appearance of FMRP in FXS neurons. Shown is the average FMRP immunofluorescence intensity per cell body in wild-type and FXS neurons. FMRP is nearly undetectable in FXS neurons but achieves levels similar to wild-type FMRP levels after 7 d of 1 µM 5-aza-dC treatment. The quantification of FMRP mean intensity was performed in *n* = 3 experiments. Shown are the mean and s.d.; univariate two-sided *t*-test.

We next asked whether 5-aza-dC reactivates *FMR1* expression in cultured FXS neurons. Since the FXS neurons are initially prepared as a mixed culture with feeder cells and undifferentiated cells, we used fluorescent in situ hybridization (FISH) to ensure that we selectively detect *FMR1* reactivation within neurons. Prior to 5-aza-dC treatment, wild-type hESC-derived neurons expressed *FMR1* mRNA, while FXS hESC-derived neurons did not, validating the specificity of the human *FMR1* FISH protocol (Figure 1A). This control demonstrates that the FISH probes do not cross react with related genes, such as *FXR1* or *FXR2*. Following 7 days of 5-aza-dC (1 µM), the overall levels of reactivated *FMR1* mRNA in 5-aza-dC-treated FXS neurons were comparable to the levels seen in wild-type neurons prior to 5-aza-dC treatment (Figure 1A,B). Overall, these experiments demonstrate that 5-aza-dC is able to restore *FMR1* mRNA expression despite the non-dividing nature of neurons.

Next, we asked whether FMRP expression was detected in 5-aza-dC-reactivated FXS neurons. Although the CGG repeat expansion in the 5′UTR *FMR1* may appear to be incompatible with translation, human chorionic villus samples from FXS embryos show the translation of *FMR1* mRNA prior to 11 weeks of gestation [4]. Additionally, *FMR1* transcripts with an expanded CGG repeat are translated in hESCs prior to differentiation [6,27]. We therefore assessed FMRP expression in FXS neurons after *FMR1* reactivation.

As expected, FMRP was readily detected by immunofluorescence in wild-type neurons, but not in FXS neurons (Figure 1A,C). These data also confirm the specificity of the FMRP immunofluorescence signal since it is only present in cells that can express FMRP. These results also confirm that the FMRP antibody does not cross react with proteins related to FMRP, such as FXR1 or FXR2. Notably, these experiments were tested in both hESC lines, further supporting the specificity of our FMRP staining. At this time point, prior to 5-aza-dC, nearly all cells are MAP2 positive, indicating that neural progenitor cells and other cell types are no longer prominent in our culture. We further confirmed that these MAP2-positive cells were post-mitotic at this time point by measuring 5-bromo-2′-deoxyuridine (BrdU, 10 µM) incorporation, which showed that almost no MAP2-positive cells were labeled with BrdU (Figure S2). Following 5-aza-dC treatment for 7 d, FXS neurons expressed similar levels of FMRP as wild-type neurons (Figure 1A,C). Thus, FMRP protein expression can be induced by 5-aza-dC treatment in neurons.

**Figure 2:**
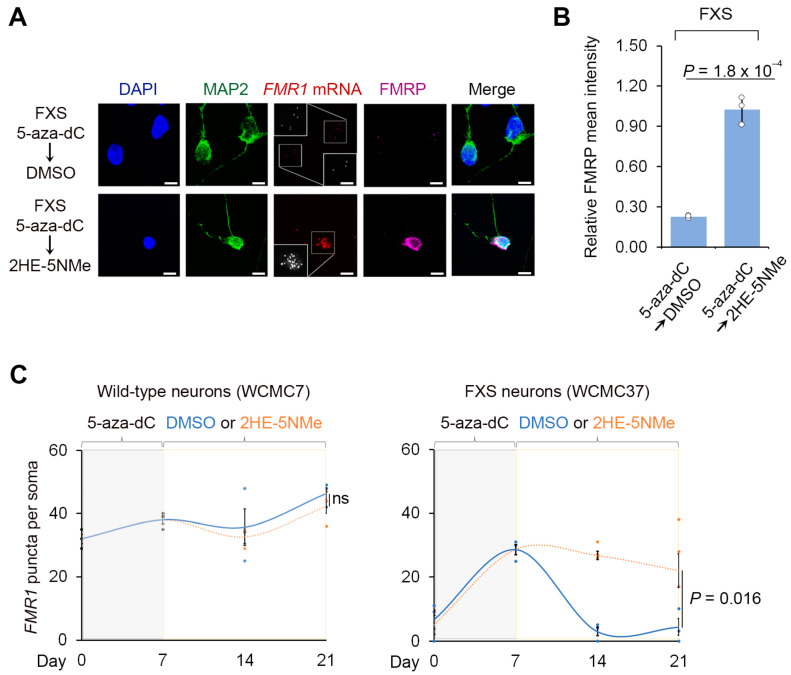
2HE-5NMe maintains *FMR1* reactivation in FXS neurons following 5-aza-dC withdrawal. (**A**) 2HE-5NMe maintains *FMR1* mRNA expression and FMRP protein levels in FXS neurons following 5-aza-dC withdrawal. Representative images (*n* = 3 experiments) of anti-MAP2 immunofluoresence (green), anti-FMRP immunofluorescence (violed), *FMR1* FISH (red) and DAPI staining (blue) of FXS neurons derived from WCMC37 hESCs treated with 1 µM 5-aza-dC for 7 days followed by 14 days of either DMSO or 500 nM 2HE-5NMe (scale bar, 10 µm). Insets show images enlarged by 150% with FISH puncta in white. (**B**) 2HE-5NMe maintains the appearance of FMRP in FXS neurons. Shown is the average FMRP immunofluorescence intensity per cell body in FXS neurons treated with 7 d of 1 µM 5-aza-dC treatment followed by 14 d of 500 nM 2HE-5NMe or DMSO. FMRP levels drop when 5-aza-dC is withdrawn and replaced with vehicle (DMSO) for 14 d. The quantification of FMRP mean intensity was performed in *n* = 3 experiments. Shown are the mean and s.d.; univariate two-sided *t*-test. (**C**) The quantification of *FMR1* FISH foci in wild-type (WCMC7, left) and FXS (WCMC37, right) neurons following 7 days of treatment with 1 µM 5-aza-dC followed by either 14 days of DMSO or 14 days of 500 nM 2HE-5NMe (*n* = 3 experiments, mean and s.d.; univariate two-sided *t*-test).

We next asked whether the reactivation of *FMR1* in post-mitotic neurons depends on the continuous presence of 5-aza-dC. We therefore switched the media to 5-aza-dC-free media after 7 d of 5-aza-dC treatment. After 14 d, the FXS neurons displayed a significant reduction in *FMR1* mRNA and FMRP protein (Figure 2 and Figure S1B). Overall, these data show that the reactivation of *FMR1* can occur in post-mitotic FXS neurons derived from human embryonic stem cells, and this reactivation is associated with increased expression of FMRP protein. However, the reactivation is dependent on the continuous presence of 5-aza-dC, and the withdrawal of 5-aza-dC results in the loss of *FMR1* expression.

### 3.2. A CGG Repeat RNA-Binding Small Molecule Maintains FMR1 Reactivation in Neurons

We next wanted to maintain the expression of *FMR1* after the removal of 5-aza-dC in FXS neurons. We previously found that 1a, a CGG repeat RNA-binding molecule, prevented the epigenetic silencing of *FMR1* in human embryonic stem cells [6]. We therefore wanted to determine if interfering with the CGG repeat RNA would block the resilencing seen in FXS neurons after 5-aza-dC withdrawal.

We first sought to develop an improved CGG repeat RNA-binding ligand. Note that 1a shows relatively low affinity for CGG repeat RNA, requiring concentrations of 10 µM for blocking CGG repeat RNA function [6]. We therefore used a higher-affinity CGG repeat RNA-binding small molecule, 2HE-5NMe, which is a dimeric version of 1a that shows >15-fold higher affinity and longer residence time on CGG repeat RNA [18] and higher specificity for RNA CGG repeat sequences compared to DNA CGG repeat sequences (Figure S3).

We next asked if 2HE-5NMe maintains *FMR1* reactivation following 5-aza-dC withdrawal. In these experiments, wild-type and FXS neurons derived from two FXS hESC lines were treated with 5-aza-dC for 7 d to reactivate *FMR1*, followed by the replacement of the media with DMSO or the CGG repeat RNA-binding small molecule. After 14 d, *FMR1* mRNA was measured by FISH in MAP2-expressing neurons. In wild-type neurons, the initial 5-aza-dC treatment or the subsequent 2HE-5NMe treatment had no significant effect on *FMR1* expression (Figure 2C). In FXS neurons, switching from 5-aza-dC to DMSO-containing media caused *FMR1* mRNA levels to return to baseline levels (Figure 2A,C). However, FXS neurons switched to media containing 2HE-5NMe (0.5 µM) showed maintenance of *FMR1* mRNA levels (Figure 2A,C). Similar results were observed in fibroblasts and lymphoblasts (Figure S4A,B). The same experiment was repeated with a second neuronal line SI-214 from FXS hESCs (Figure S4C).

We next wanted to compare the *FMR1* mRNA-maintenance effects of 2HE-5NMe to epigenetic pathway inhibitors that affect pathways linked to the formation of the repressive marks at the *FMR1* locus. We first tested several small molecule inhibitors of pathways linked to *FMR1* gene silencing, including GSK343, which inhibits the H3K27-methylating enzyme EZH2, and histone deacetylase inhibitors suberoylanilide hydroxamic acid (SAHA), splitomycin and trichostatin A (TSA) [11,12,28,29]. Each of these compounds was applied after the withdrawal of 5-aza-dC on day 7. Of these inhibitors, only GSK343 prevented complete loss of *FMR1* mRNA levels after the withdrawal of 5-aza-dC (Figure S4D,E). However, 2HE-5NMe was markedly more effective than any of these epigenetic pathway inhibitors. Overall, these data suggest that 2HE-5NMe is highly effective in maintaining *FMR1* expression in FXS neurons.

We also asked if 2HE-5NMe exhibits toxicity, which may account for its ability to maintain *FMR1* mRNA expression. However, RNA-Seq analysis of vehicle and 2HE-5NMe-treated neurons showed no major transcript differences or activation of genes linked to cell toxicity (Figure S5).

We next asked whether 2HE-5NMe maintains expression of FMRP in FXS neurons. FXS neurons were treated with 5-aza-dC for 7 d and then switched to media containing DMSO or 2HE-5NMe. In FXS neurons switched to DMSO-containing media, FMRP immunofluorescence staining was not detected in neurons (Figure 2A,B). In contrast, FXS neurons switched to 2HE-5NMe-containing media retained FMRP in the absence of 5-aza-dC (Figure 2A,B). Overall, these data indicate that 2HE-5NMe can be used to maintain the expression of FMRP protein in FXS neurons that would otherwise lack FMRP.

### 3.3. 2HE-5NMe Selectively Maintains Epigenetic Activation of theFMR1 Locus

A problem with most small molecules that target epigenetic regulators is that they perturb histone modifications throughout the genome, thus affecting the expression of large numbers of genes. Since 2HE-5NMe selectively binds CGG repeat RNA, we reasoned that 2HE-5NMe may allow relatively selective control of the epigenetic state of *FMR1*. To test this, we monitored the genome-wide distribution of H3K27me3, a repressive mark that has been detected broadly throughout the silenced *FMR1* gene locus in both FXS patients and FXS-derived cell lines [11]. We also monitored H3K4me3, a marker of gene activation that is lost at the silenced *FMR1* gene in FXS cell lines [30]. H3K4me3 and H3K27me3 were utilized as representative markers for active or repressed promoters, respectively.

**Figure 3:**
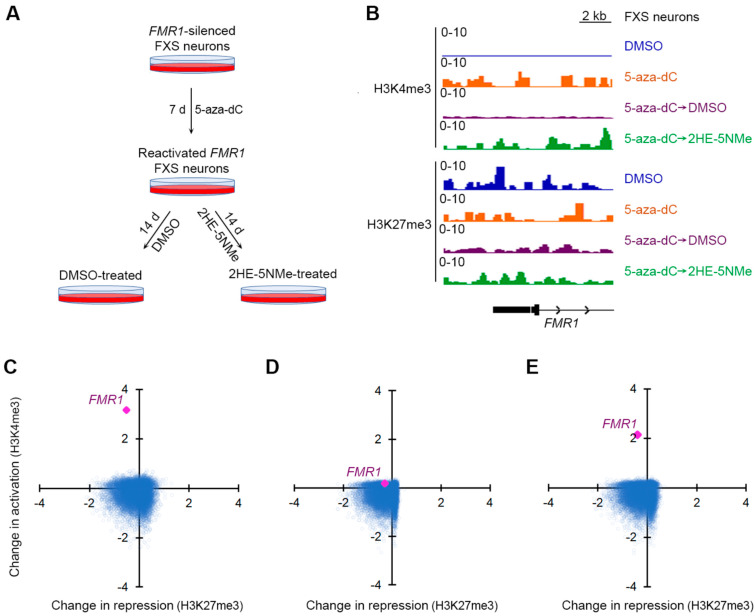
5-aza-dC induces H3K4me3 at the *FMR1* locus, which is selectively maintained by 2HE-5Nme. (**A**) A schematic depiction of 5-aza-dC treatments followed by 2HE-5Nme-mediated maintenance of *FMR1* gene activation. FXS neurons were treated with 5-aza-dC for 7 d to reactivate the *FMR1* allele, followed by replacement in media lacking 5-aza-dC and containing either DMSO (vehicle) or 2HE-5Nme for 14 d to prevent the resilencing of the *FMR1* allele. (**B**) The effect of 5-aza-dC and 2HE-5Nme on activating and repressive histone marks at the *FMR1* promoter. Neurons were treated with 5-aza-dC (1 µM, 7 d), which was then replaced with either DMSO or 2HE-5Nme (14 d). Histone marks were measured using CUT&RUN. The activating mark H3K4me3 is largely absent on the 5′ region of the *FMR1* gene in untreated FXS neurons (DMSO), but it is markedly induced in 5-aza-dC-treated neurons. This signal is nearly completely depleted in FXS neurons switched to DMSO for 14 d, but it remains detected in FXS neurons switched to 2HE-5Nme (500 nM). The repressive mark H3K27me3 is readily detected on the *FMR1* locus in all conditions. (**C**) Shown is a scatter plot indicating the change in H3K4me3 and H3K27me3 as measured by CUT&RUN for gene promoters in FXS neurons treated with 5-aza-dC relative to untreated FXS neurons. *FMR1* is the most prominently affected gene after 5-aza-dC treatment, and it shows a marked increase in H3K4me3 levels. (**D**) The withdrawal of 5-aza-dC and culturing in DMSO leads to the loss of H3K4me3 marks at the *FMR1* promoter. Here, we compared histone marks in neurons cultured in 5-aza-dC and then DMSO to untreated FXS neurons. The scatter plot is prepared as in *C*. (**E**) The withdrawal of 5-aza-dC and culturing in 2HE-5Nme maintains H3K4me3 marks at the *FMR1* promoter. Here, we compared histone marks in FXS neurons cultured in 5-aza-dC and then 2HE-5NMe (500 nM) to untreated FXS neurons. The scatter plot is prepared as in *C*.

To measure these active and repressive marks, we used CUT&RUN, a genome-wide mapping technique [31] that is optimal for smaller cell populations, such as the number of neurons generated from human embryonic stem cells. In the CUT&RUN protocol, cells are permeabilized and incubated with antibodies that selectively recognize the histone marks. The DNA associated with the histones and bound antibodies are liberated enzymatically for the sequencing and localization of active and repressive marks across the genome. CUT&RUN was performed for untreated FXS neurons, FXS neurons treated with 5-aza-dC for seven days, FXS neurons treated with 5-aza-dC for seven days and then DMSO for fourteen days and FXS neurons treated with 5-aza-dC for seven days and then 2HE-5NMe for fourteen days (Figure 3A). Reproducibility was compared between runs and was determined to be high (Pearson = 0.944) (Figure S6A).

First, we asked which gene promoters showed changes in active or repressive marks following 5-aza-dC treatment. We used CUT&RUN to compare active and repressive marks before and after 5-aza-dC treatment. Following 5-aza-dC treatment, the active mark H3K4me3 showed markedly increased reads that mapped to the *FMR1* promoter (Figure 3B). However, there was only a small drop in the H3K27me3 repressive mark (Figure 3B). This agrees with a previous study that found that 5-aza-dC induces a larger change in activation marks than repressive marks at the *FMR1* locus in FXS lymphoblastoid cells [32].

We next examined the effect of 5-aza-dC across the genome of FXS neurons. Compared to other gene promoters, *FMR1* has a more substantial fold increase in activation marks than any other gene (Figure 3C and Figure S6B) in 5-aza-dC treated FXS neurons relative to untreated FXS neurons. This likely reflects the low basal level of activation marks in FXS neurons prior to 5-aza-dC treatment.

Next, we sought to understand the transcriptome-wide distribution of histone marks after the withdrawal of 5-aza-dC. After replacing 5-aza-dC-containing media with DMSO-containing media for 14 d, H3K4me3 were lost from the *FMR1* promoter, resulting in similar H3K4me3 levels to those observed in untreated FXS neurons (Figure 3D and Figure S6C).

Finally, we examined the transcriptome-wide distribution of histone marks after 5-aza-dC was replaced with 2HE-5NMe for 14 d. Here, the *FMR1* promoter maintained the selective expression of active marks (Figure 3E and Figure S6D). The gene ranking of changes in activation marks at promoters suggest that 5-aza-dC specifically increases histone activation marks at the promoter of *FMR1* and that 2HE-5NMe specifically maintains those marks (Figure S6B–D). Overall, treatment with 5-aza-dC and 2HE-5NMe causes a selective activation of the *FMR1* gene, and 2HE-5NMe does not appear to cause off-target epigenetic activation on other genes in the transcriptome.

### 3.4. 2HE-5NMe Reverses Dendritic Spine Abnormalities in FXS Neurons

**Figure 4:**
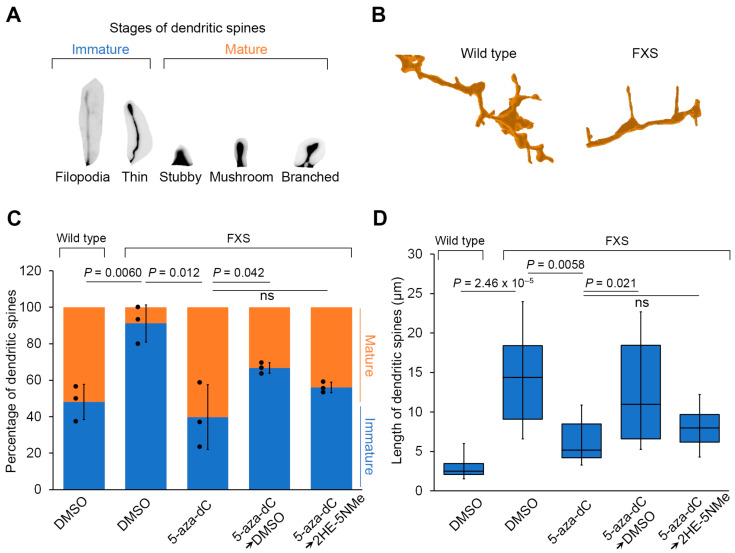
Transient 5-aza-dC treatment induces dendritic spine maturation, which can be maintained by 2HE-5NMe. (**A**) A schematic depicting different stages of dendritic spine maturation. Immature spines are common in FXS and are recognized by their long and thin morphology. (**B**) FXS neurons have a higher percentage of immature spines compared to wild-type neurons. FXS neurons are derived from WCMC37 hESCs. Representative images of 3D reconstructed dendritic spines from wild-type and FXS neurons. Long and thin spines are readily detected in FXS neurons. (**C**) The high prevalence of immature dendritic spine maturation in FXS neurons can be reversed by treatment with 1 µM 5-aza-dC for 7 d and maintained replacement with media containing 500 nM 2HE-5NMe for 14 d. 5-aza-dC treatment results in the appearance of mature spines and the loss of immature spines and thus resembles wild-type neurons. Switching to DMSO-containing media causes the number of immature spines to increase and a loss of mature spines. In contrast, 2HE-5NMe (500 nM) improves the maintenance of mature spines. The quantification of the percentage of immature versus mature dendritic spines in wild-type neurons or FXS neurons following treatments was performed in *n* = 3 experiments. Shown are the mean and s.d.; univariate two-sided *t*-test. (**D**) The spine length of dendritic spines in FXS neurons shortens when the neurons are treated with 1 µM 5-aza-dC for 7 d, which is indicative of dendritic spine maturation. 2HE-5Nme maintains spine maturation induced by 5-aza-dC. The quantification of dendritic spine length (μm) in FXS neurons following the indicated treatments was performed in *n* = 3 experiments (box limits, interquartile range; whiskers, minimum to maximum; center line, median; univariate two-sided *t*-test).

The classic phenotypic abnormality of FXS neurons is impaired dendritic spine maturation. This is manifested by long and thin filopodia-like dendritic spines consistent with immature spines [5]. We therefore asked whether the restoration of FMRP expression to FXS neurons using 5-aza-dC followed by 2HE-5NMe would result in dendritic spine maturation. For these experiments, we categorized protrusions on the dendrites as immature (filopodia or thin protrusions) versus mature spines (stubby, mushroom or branched) (Figure 4A). In the FXS neurons, long and thin protrusions and filopodia-like structures were highly prevalent, consistent with immature dendritic spines. Furthermore, FXS neurons had few stubby spines and did not seem to progress into the pinching phase to produce mushroom spines (Figure 4B,C). This contrasts with wild-type neurons, which exhibited markedly more mature spine-like morphologies. Overall, the average length of these protrusions in FXS neurons was significantly longer than in wild-type neurons (Figure 4D). Thus, the classic FXS-associated dendritic spine abnormalities can be seen in neurons differentiated from hESCs.

We next asked whether FMRP expression induced by 5-aza-dC and maintained by 2HE-5NMe rescues these dendritic spine abnormalities. Following 5-aza-dC treatment for 7 d, dendritic spines in FXS neurons showed evidence of pruning (i.e., shortening) and maturation (Figure 4B–D). Additionally, FXS neurons showed markedly more mushroom spines following 5-aza-dC treatment (Figure S7A). The spines in FXS neurons following 5-aza-dC treatment were also significantly shorter in length (Figure 4D). Importantly, these effects were not seen in wild-type neurons, which already exhibited a higher degree of mature spines (Figure 4C and Figure S7B). This indicates that the effects of 5-aza-dC were not due to nonspecific effects on spines.

Finally, we asked whether 2HE-5NMe maintains dendritic spine maturation in FXS neurons after 5-aza-dC is removed. After FXS neurons were switched from 5-aza-dC to media containing 2HE-5NMe for 14 d, the dendritic spines of FXS neurons showed markedly more mature spines than FXS neurons switched to DMSO-containing media (Figure 4C). Additionally, 2HE-5NMe-treated FXS neurons showed markedly shorter spines compared to spines in neurons switched to DMSO-containing media (Figure 4D). Thus, the removal of 5-aza-dC results in the reversal of dendritic spine maturation in FXS neurons, and this effect can be blocked by treatment with 2HE-5NMe.

## 4. Discussion

In this study, we show that the epigenetically silenced state of *FMR1* in FXS can be reversed and maintained in an active state in diverse cell types, including neurons. This approach relies on 5-aza-dC, a nonspecific epigenetic reactivator which reactivates *FMR1* in FXS. Although 5-aza-dC reactivates the *FMR1* allele, continuous administration may be problematic in patients due to toxicity and poor pharmacokinetics of 5-aza-dC [33]. We describe an approach involving a brief treatment with 5-aza-dC followed by the maintenance of *FMR1* activation with 2HE-5NMe, an RNA-binding small molecule. 2HE-5NMe binds the CGG repeat portion of the *FMR1* mRNA, thus selectively blocking the resilencing of the *FMR1* gene. 2HE-5NMe maintains *FMR1* gene activation and allows continuous FMRP protein production in neurons. Genome-wide profiling of active and repressive histone marks shows that 2HE-5NMe maintains the active state of the *FMR1* allele without inducing the activation of other genes. *FMR1* reactivation in neurons results in the re-expression of FMRP and reversal of neuronal morphology defects that are characteristic of FXS neurons. Overall, this approach demonstrates that an RNA-binding small molecule can be used for gene-specific epigenetic control, and it provides an approach for the restoration of FMRP expression in FXS neurons.

Our experiments are designed to test the concept of selective epigenetic reactivation. 2HE-5NMe should not be viewed as a compound that is ready for use in humans since it has not undergone thorough medicinal chemistry to remove off-target effects. Indeed, as with any molecule that has not been rigorously optimized to minimize off-target binding, diverse effects on gene expression are very likely. For this reason, we used CUT&RUN to detect specific epigenetic changes regulated by 2HE-5NMe. These studies, along with RNA-seq experiments, support the idea that 2HE-5NMe shows a high degree of selectivity towards the reaction of *FMR1*. Additional studies will be needed to address pharmacokinetics, off-target effects and *in vivo* efficacy, especially in humans. Thus, the results presented here provide motivation to develop high-affinity and high-selectivity compounds with desirable pharmacokinetics for further testing in preclinical FXS models.

The ability of 2HE-5NMe to selectively control the epigenetic state of *FMR1* reflects its ability to bind CGG repeat RNA, which are uniquely expanded in the *FMR1* allele. The CGG repeats are the pathogenic mediators of epigenetic silencing with the ability to form R-loops with the complementary region of the *FMR1* gene, as well the ability to form RNA secondary structures, which can act as ribonucleoprotein-binding sites [34,35,36]. By binding to the CGG repeat sequence, small molecules can disrupt the ability of the CGG repeat RNA from interacting with DNA or proteins [6]. Despite the ability of 2HE-5NMe to bind the CGG repeat sequence in the 5′UTR of the *FMR1* mRNA, the translation of the *FMR1* mRNA is not blocked. Thus, 2HE-5NMe can block the silencing function of the CGG repeat without impairing the translation of the *FMR1* mRNA.

Notably, the reactivation of the *FMR1* allele is not sufficient to produce FMRP protein in all cell types. In FXS fibroblasts, we found that treatment with 5-aza-dC resulted in *FMR1* mRNA expression without FMRP expression, consistent with previous studies in lymphoblastoid cells [10]. This presumably reflects impaired ribosome scanning through the CGG repeat expansion in these cells. In contrast to lymphoblastoid cells, the reactivation of *FMR1* in other cell types can lead to FMRP synthesis despite the CGG repeat, including neural progenitor cells [37]. Additionally, FMRP is detected in FXS hESCs prior to differentiation [6,27] and in chorionic villus samples from human embryos with FXS prior to 11 weeks of gestation [4]. Thus, in some cell types, the ribosome can scan past the CGG repeat, presumably due to the expression of specific helicases that may unwind the CGG repeat structures. Alternatively, internal mRNA entry mechanisms by the ribosome may be operational in these cells regardless of the precise CGG repeat. This raises the possibility that the reactivation of *FMR1* with 5-aza-dC followed by the maintenance of *FMR1* activity with 2HE-5NMe may be able to restore FMRP to FXS neurons.

Notably, even small amounts of FMRP protein can be sufficient for normalizing neuronal function. Recent studies using a CRISPR-based reactivation approach found that as little as 5% of basal levels of FMRP is sufficient to restore the increased levels of the spontaneous activity of FXS neurons [38]. Thus, *FMR1* reactivation approaches, even if inefficient, might confer normal phenotypes to FXS neurons. It will be important to test whether *FMR1* reactivation and FMRP expression restores behavioral phenotypes, rather than only neuronal morphology. Comprehensive behavioral and electrophysiological assays will be important to ultimately assess improved neurological function. Newer approaches using the engraftment of human FXS neurons in the mouse brain [30] are ideally suited for testing the ability of reactivated FXS neurons to regulate neuronal circuit function. These models can also be used to assess the off-target activity and durability of effects during the long-term administration of 2HE-5NMe. Although we used a single dose of 5-aza-dC, patients may require intermittent doses of 5-aza-dC to reactivate *FMR1* if the gene becomes silenced over time. The mouse models with engrafted human FXS neurons will be important to reveal how long 5H-NMe can be used before the *FMR1* locus eventually becomes resilenced and another 5-aza-dC treatment would be needed.

A surprising finding was that 5-aza-dC can reactivate *FMR1* in neurons, even though neurons are not dividing cells. 5-aza-dC has been used to show *FMR1* reactivation in mixed cultures of neuronal progenitor cells and neurons derived from FXS induced pluripotent stem cells (iPSC) [39]. However, it is unclear whether *FMR1* reactivation was occurring within neurons or in mitotic neuronal precursor cells prior to neuronal differentiation [40]. In our study, 5-aza-dC was added to cells after neurons acquired their neuronal identity, thus demonstrating that 5-aza-dC-mediated *FMR1* reactivation can occur in post-mitotic cells. We confirmed that our neurons expressed MAP2, a marker of differentiated neurons [41,42,43]. Our experiments suggest that these MAP2-positive cells rarely showed evidence of cell division. By looking at MAP2-positive cells specifically, we ensured that we were almost completely looking at post-mitotic neurons in our immunofluorescence and FISH assays that were used to confirm *FMR1* and FMRP expression.

The mechanism of 5-aza-dC in the FXS neurons described here is unclear. Repeat sequences can undergo transient DNA helix destabilization, causing extrahelical extrusions. These are recognized by DNA mismatch repair pathways, which typically include DNA nicking, followed by excision and resynthesis of DNA [44,45]. Thus, 5-aza-dC can potentially incorporate into the CGG repeat region of *FMR1* during DNA damage and repair. The incorporation of 5-aza-dC would then make the CGG repeat region resistant to methylation. Notably, CGG repeat damage and subsequent repair have been observed in oocytes, during their non-dividing phases [46]. Thus, the expanded CGG repeat appears to be a site of enhanced DNA damage, even in non-dividing cells.

The idea that 5-aza-dC can affect post-mitotic neurons is supported by multiple recent studies that show that 5-aza-dC affects gene expression in post-mitotic neurons [15,16]. This includes genes encoding RNAs with disease-associated repeats [17,47]. Thus, repeat-expanded sequences may be sensitive to 5-aza-dC.

5-aza-dC may also function through pathways that do not involve DNA synthesis. Previous studies have shown that 5-aza-dC inhibits thymidylate synthase, which can lead to alterations in the global nucleotide pool [48,49]. This mechanism may affect epigenetic silencing of *FMR1* through currently unknown mechanisms.

An important question is whether the reactivation of *FMR1* expression and the subsequent maintenance of gene expression by 2HE-5NMe is due to the contraction of the CGG repeat expansion. Contraction would be a permanent reactivation of *FMR1*. Our experiments show that reactivation is reversible by the removal of 5-aza-dC. Thus, in this study, as well as our previous work with FXS hESCs [6], the return of silencing upon the withdrawal of either 5-aza-dC or CGG repeat-binding molecules demonstrates that repeat contraction is an unlikely mechanism for *FMR1* expression in our cells. Instead, the mechanism of 5-aza-dC likely involves reversal of the silencing epigenetic marks.

It should be noted that the FXS hESC lines used in this study are greater than 200 repeats^2^. Consistent with this, we found essentially no FMRP-positive neurons prior to 5-aza-dC treatment, further indicating that few FXS hESCs had undergone repeat contraction to under 200 repeats while they were cultured. Overall, epigenetic reactivation rather than repeat contraction more clearly explains our findings.

Our studies raise the possibility that 5-aza-dC can be used therapeutically with a CGG repeat-binding small molecule to reactivate and maintain *FMR1* mRNA and FMRP protein expression. Although our studies utilized 2HE-5Nme, this compound is a tool compound and has not been optimized for use as a therapeutic. Nevertheless, our work points to the potential value in developing highly potent CGG repeat-binding small molecules. Further development should focus on minimizing any pharmacologically relevant off-target effects and optimizing the affinity, selectivity, blood–brain permeability and pharmacokinetic and pharmacodynamic properties needed for a medical therapeutic.

## Data Availability

Sequencing data for the CUT&RUN experiment have been deposited in NIH Gene Expression Omnibus (*GEO*) database GSE272191, while the RNA-seq data have been deposited in GSE272192.

## Supplementary Materials

The following supporting information can be downloaded at: https://www.mdpi.com/article/10.3390/genes16030278/s1, Figure S1: Time course of 5-aza-dC treatment and withdrawal and effects on FMR1 mRNA and FMRP levels A) Determination of the lowest concentration of 5-aza-dC to reactivate FMR1 mRNA expression in FXS fibroblasts (Coriell cell line GM07072; Figure S2: MAP2-labeled cells are largely non-dividing, differentiated cell; Figure S3: Measurement of binding affinity (dissociation constants) for 1a and 2HE-5NMe to various nucleic acid structures; Figure S4: 5-aza-dC induces FMR1 mRNA levels which is maintained by 2HE- 5NMe in different FXS cell types; Figure S5: 2HE-5NMe does not alter global gene expression; Figure S6: CUT&RUN runs are highly reproducible and indicate the specificity of 5-aza-dC and 2HE-5NMe for FMR1; Figure S7: Defective FXS dendritic spine maturation can be restored with drug treatments.
