# Supplementary material for: Sustained Epigenetic Reactivation in Fragile X Neurons with an RNA-Binding Small Molecule
Source: Genes (Basel). 2025 Feb 25;16(3):278. doi: 10.3390/genes16030278 (PMC11942054; doi:10.3390/genes16030278)
Supplement: Supplementary file 1 [file genes-16-00278-s001.zip › genes-3463055-supplementary.pdf]

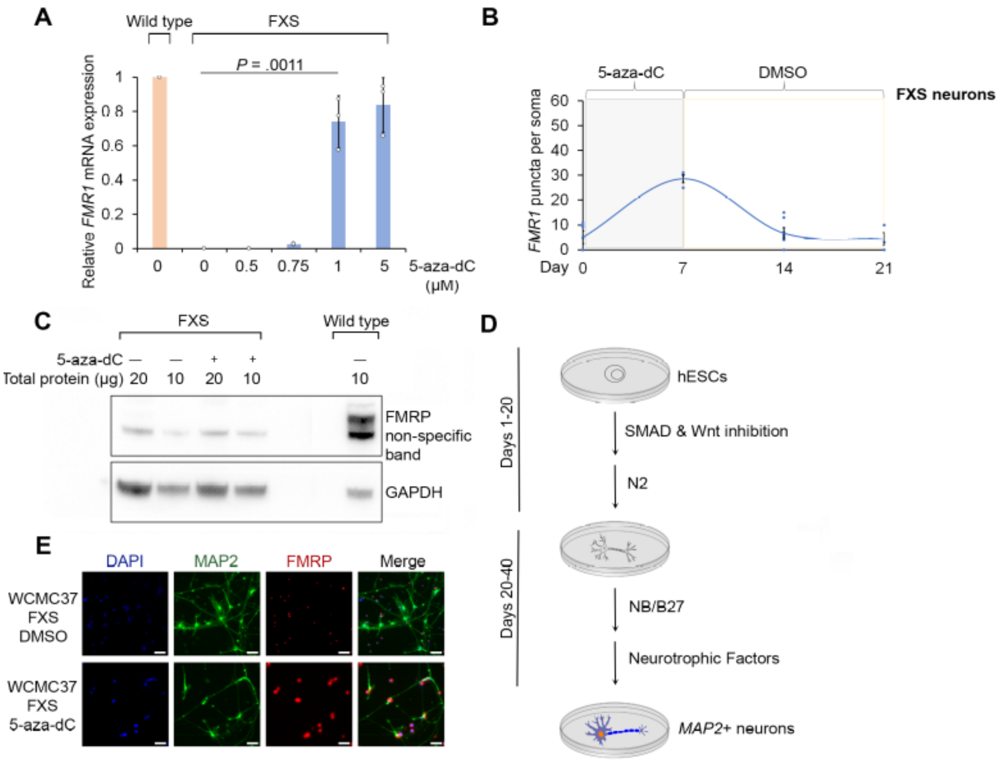

Supplemental Figure S1. Time course of 5-aza-dC treatment and withdrawal and effects on *FMR1* mRNA and FMRP levels *A*) Determination of the lowest concentration of 5-aza-dC to reactivate *FMR1* mRNA expression in FXS fibroblasts (Coriell cell line GM07072). Titration analysis shows that 1 µM 5-aza-dC is the lowest amount of 5-aza-dC that significantly reactivates *FMR1*. For subsequent experiments, 1 µM 5-aza-dC was used. Quantification of the amount of *FMR1* mRNA in FXS fibroblasts was performed using qRT-PCR (see Methods) following treatment with the indicated concentrations of 5-aza-dC for 7 d (n = 3 experiments, mean and s.d.; univariate two-sided t-test). (*B*) A time-course to determine the persistence of *FMR1* reactivation in FXS fibroblasts (GM07072) after withdrawal of 1 µM 5-aza-dC. *FMR1* mRNA was measured using qRT-PCR at 14 and 21 d (which represents 7 and 14 d after withdrawal of 5-aza-dC, respectively). *FMR1* mRNA nearly returned to baseline levels after 7 days, and completely returned to basal levels at 14 d. For subsequent experiments, we measured resilencing at 14 d after withdrawal of 5-aza-dC (n = 3 experiments, mean and s.d.; univariate two-sided t-test). (*C*) FXS fibroblasts (GM07072) treated with 5-aza-dC do not express FMRP. FMRP was measured by western blotting. Note that only the upper band (labeled FMRP) is the specific FMRP band. FMRP is only detected in wild-type fibroblasts (MRC5), and not in FXS fibroblasts, even after 5-aza-dC treatment. (*D*) Schematic depicting the neuronal differentiation method using human embryonic stem cells (hESC). NB/B27 refer to culture media. (*E*) FXS neurons (derived from WCMC37 hESC) express FMRP following treatment with 5-aza-dC. In the main figure (Fig. 2A), high power images showing a single neuron is presented to allow visualization of *FMR1* puncta and FMRP localization. Here we show representative lower power objective images (n = 3 experiments) with multiple neurons

per field of view. Shown is anti-MAP2 and anti-FMRP immunofluorescence staining of FXS neurons that were untreated or treated with 5-aza-dC for 7 days (scale bar, 50 μM). FMRP is readily detected after 5-aza-dC treatment.

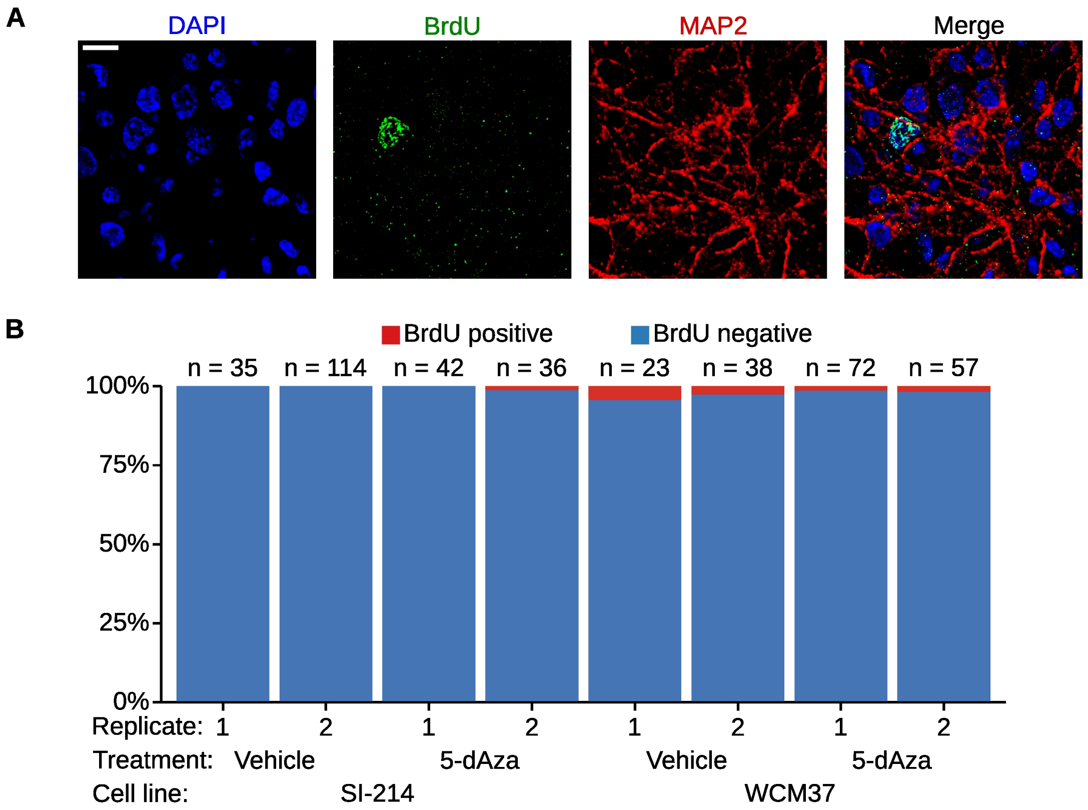

Supplemental Figure S2. MAP2-labeled cells are largely non-dividing, differentiated cells. To determine if MAP2 neurons are terminally differentiated, non-dividing neurons during our 5-aza-dC treatment, we used BrdU staining. Neurons were differentiated from either SI-214 or WCM37 hESCs using the protocol outlined in Supplementary Figure S1D. These neurons were then treated with BrdU (10 µM) with or without 5-aza-dC (1 µM) for 48 hr. BrdU and MAP2 were detected using immunofluorescence and DNA was labeled using DAPI.

A) WCM37 cells treated with 5-aza-dC for 48 h. Immunofluorescence was then performed using DAPI (blue), anti-BrdU antibodies (green) and MAP2 antibodies (red). Scale bar, 10 µm.

B) Quantification of the number of dividing cells labeled with MAP2 after 48 hr of BrdU treatment in each biological replicate. For each replicate, cells were imaged on the basis of MAP2 signal in 5-10 z stacks. The percentage of MAP2 cells in each Z stack stained with BrdU (y axis, red) and the percentage that were not stained with BrdU (y axis, blue) are plotted for each replicate of SI-214 (left) and WCM37 cells (right) with or without 5-aza-dC treatment (x-axis). Only a small fraction of MAP2+ cells divided after the start of 5-aza-dC treatment, suggesting that cell division is unlikely to be required for the effect of 5-aza-dC on *FMR1* expression.

A

B

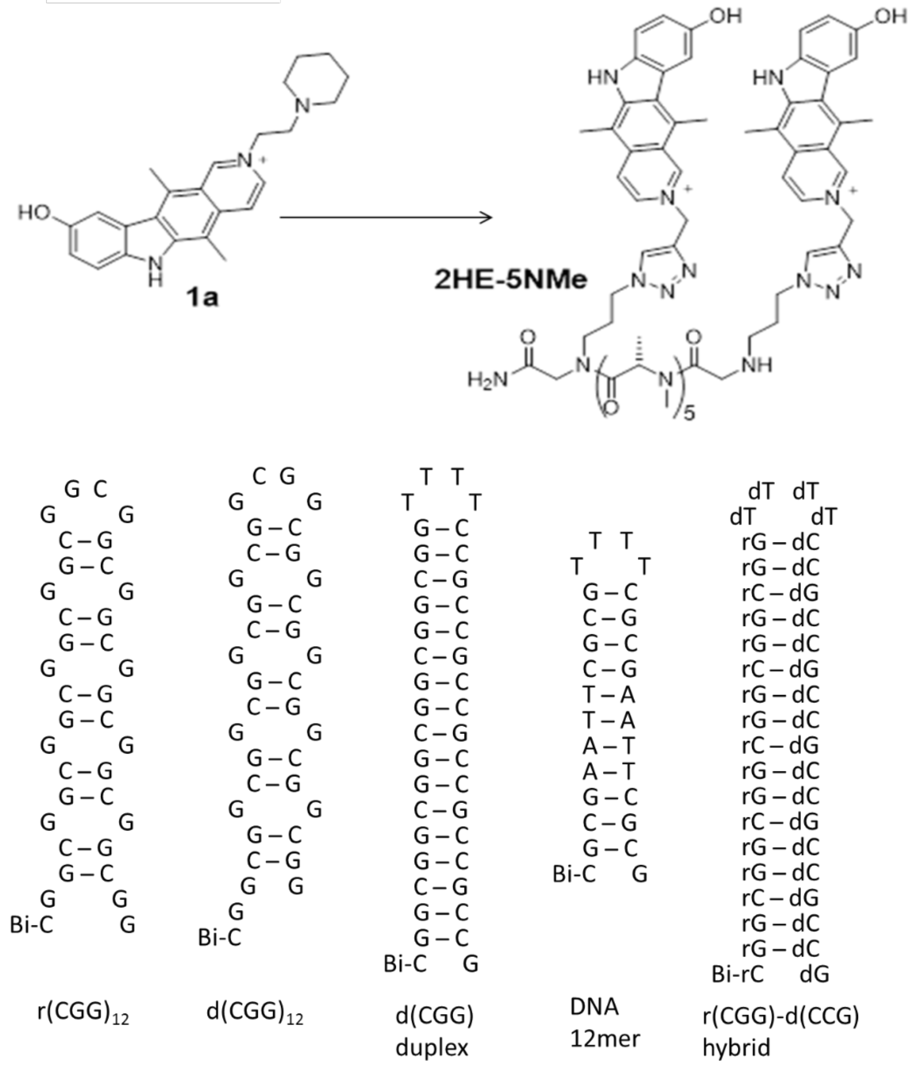

**Compound**	**1a**			**2HE-5NMe**		
Nucleic acid	r(CGG)$_{12}$	r(CGG)$_{12}$	d(CGG)$_{12}$	d(CGG) duplex	DNA 12mer	r(CGG)-d(CCG)
K_d, M	8.00e-7	5.00e-8	1.41e-6	6.72e-6	6.51e-6	4.21e-6
	5.50e-5	6.50e-8	1.75e-6	4.81e-5	2.41e-5	1.20e-5

Supplemental Figure S3. Measurement of binding affinity (dissociation constants) for 1a and 2HE-5NMe to various nucleic acid structures. *A)* Structures of compounds 1a and 2HE-5NMe. *B)* At the top are indicated the predicted structures of 12 CGG repeats as RNA, 12 CGG repeats as DNA, a double-stranded DNA-structure containing 12 CGG repeats, a random DNA 12mer, and a RNA-DNA hybrid of 12 CGG RNA repeats to 12 CGG DNA repeats. Below are indicated the dissociation constants as measured by biolayer interferometry.

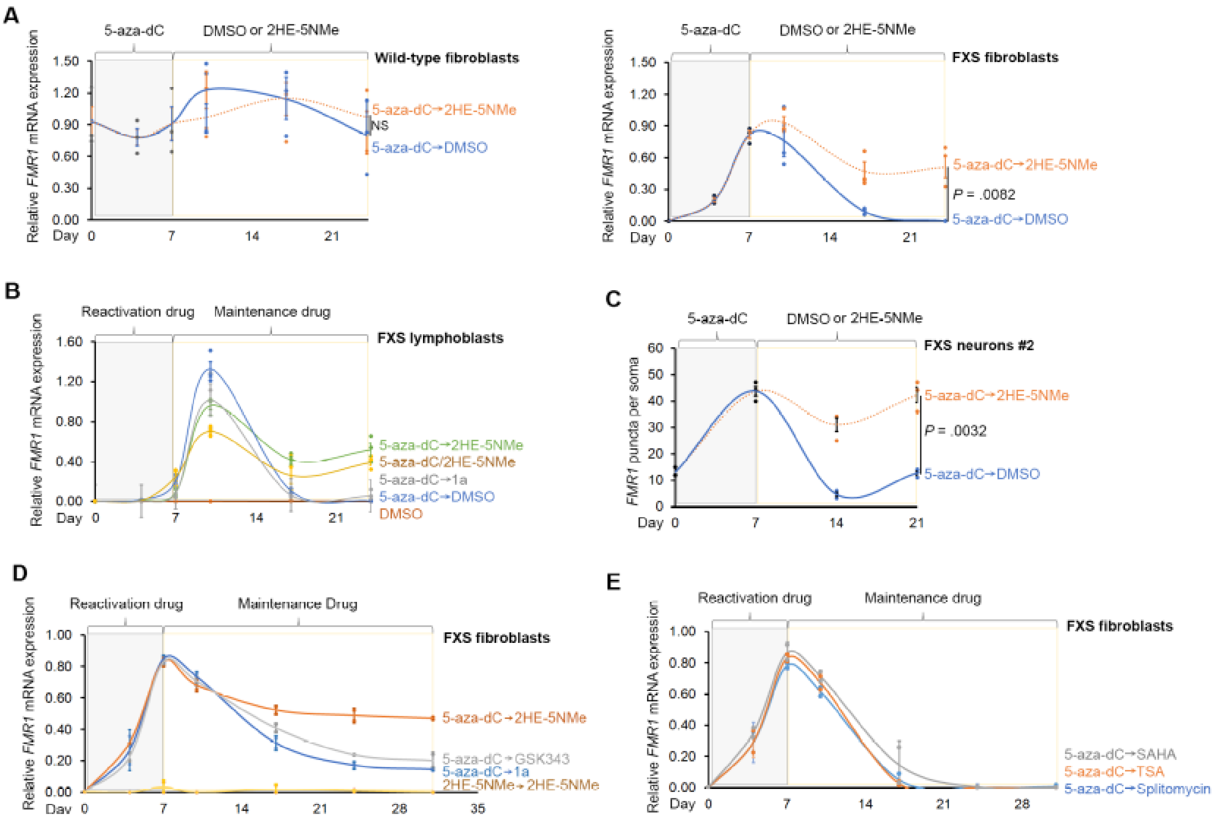

Supplemental Figure S4. 5-aza-dC induces *FMR1* mRNA levels which is maintained by 2HE-5NMe in different FXS cell types. (*A*) 5-aza-dC reactivates *FMR1* mRNA expression in FXS fibroblasts (GM07072) and 2HE-5NMe maintains *FMR1* mRNA levels after withdrawal of 5-aza-dC. Quantification of *FMR1* mRNA by qRT-PCR in wild-type (left) and FXS (right) fibroblasts following 7 d of treatment with 5-aza-dC (1 µM) followed by either 14 days of DMSO or 14 days of 2H-NMe. Wild-type fibroblasts (MRC5) do not show a substantial effect of any treatment on *FMR1* mRNA levels. In contrast, FXS fibroblasts show *FMR1* mRNA reactivation in response to 5-aza-dC, which can be maintained by 500 nM 2HE-5NMe. *n* = 3 experiments, mean and s.d.; univariate two-sided *t*-test. (*B*) 5-aza-dC reactivates *FMR1* mRNA expression in FXS lymphoblasts (GM06852) and 2HE-5NMe maintains *FMR1* mRNA levels after withdrawal of 5-aza-dC. Quantification of the amount of *FMR1* mRNA by qRT-PCR in FXS lymphoblasts following 5-aza-dC and various maintenance drugs (2H-5NMe, 1a, DMSO) as compared to the amount of *FMR1* mRNA in untreated wild-type lymphoblasts (GM06890, *n* = 3 experiments; mean and s.d.). As a control, FXS lymphoblasts were also treated with DMSO for duration of the entire experiment. As another control, FXS lymphoblasts were treated simultaneously with 5-aza-dC and 2HE-5NMe for the entire time course. However, this synchronous treatment (yellow line) did not show improved *FMR1* mRNA reactivation compared to sequential treatment (green line). (*C*) Quantification of *FMR1* FISH foci in a second FXS neuronal line (SI-214) following 7 days of treatment with 1 µM 5-aza-dC followed by either 14 days of DMSO or 14 days of 500 nM 2HE-5NMe (*n* = 3 experiments, mean and s.d.; univariate two-sided *t*-test). (*D*) 2HE-5NMe is more efficient than epigenetic silencing inhibitors at maintaining 5-aza-dC-induced reactivation of *FMR1* mRNA. Quantification of the amount of *FMR1* mRNA in FXS fibroblasts

(GM07072) following treatment with 1 μM 5-aza-dC (7 d), followed by 5-aza-dC removal and treatment with various drugs to maintain *FMR1* expression (500 nM 2H-5NMe, 2.5 μM GSK343, 10 μM 1a). *FMR1* FISH signals were normalized to the levels of *FMR1* mRNA in untreated wild-type fibroblasts. As a control, FXS fibroblasts are also treated with 2HE-5NMe without 5-aza-dC for the entire time course, without 5-aza-dC. No *FMR1* reactivation is seen. *FMR1* mRNA levels were quantified by FISH in *n* = 2 experiments; Shown are mean and s.d. (*E*) Histone deacetylase inhibitors are unable to maintain expression of *FMR1* mRNA expression following withdrawal of 5-aza-dC. Quantification of the amount of *FMR1* mRNA in FXS fibroblasts (GM07072) following treatment with 5-aza-dC and then replacement with various epigenetic pathway inhibitors that may prevent epigenetic silencing of the *FMR1* locus (SAHA, TSA, splitomycin). *FMR1* mRNA levels in the treated FXS fibroblasts are compared to the amount of *FMR1* mRNA in untreated wild-type fibroblasts (*n* = 2 experiments; mean and s.d.). As can be seen, these compounds did not prevent resilencing of *FMR1* mRNA expression. This contrasts with 2HE-5NMe, as shown in Supplemental Fig. S4C.

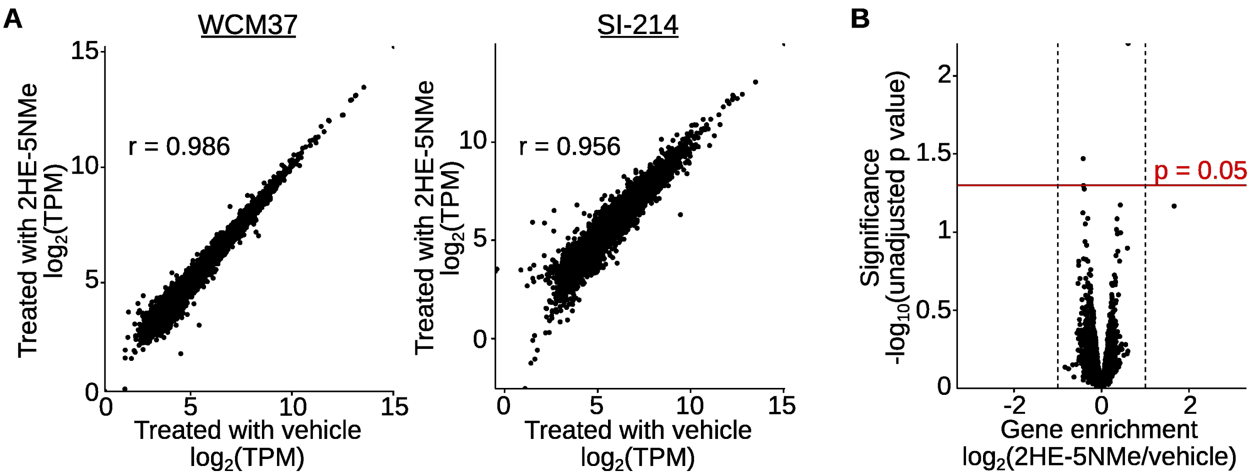

Supplemental Figure S5. 2HE-5NMe does not alter global gene expression. To determine if 2HE-5NMe is specific, RNA-seq was performed on SI-214 and WCM37 cells that were treated with 2HE-5NMe or vehicle for two weeks. Each set of hESCs was treated using the neuron differentiation protocol described in Fig. S1D and then treated with 5-aza-dC (1 µM) for 7 days. The cells were then treated with 2HE-5NMe or vehicle for 14 days. RNA-seq was then performed on each sample.

A) Gene expression is highly correlated between cells treated with vehicle and 2HE-5NMe. Expression of mRNA in WCM37 (left) and SI214 (right) cells is plotted for each gene, with expression after treatment with vehicle (x-axis) plotted against expression after treatment with 2HE-5NMe (y-axis). The Pearson's correlation coefficient (r) is plotted for each cell type, n = 2.

B) To examine whether 2HE-5NMe induces significant changes in the expression of any genes, we used DESeq2 to calculate the significance of fold change differences. Here, for each gene, the fold-change difference in RNA expression in 2HE-5NMe samples relative to vehicle (x-axis) is plotted against the unadjusted significance of the fold-change difference (y-axis). The unadjusted p value = 0.05 is plotted in red. While a couple of genes have an unadjusted $p < 0.05$, no genes have an $FDR < 0.05$. Thus, no genes have significantly changed expression after two weeks of 2HE-5NMe treatment.

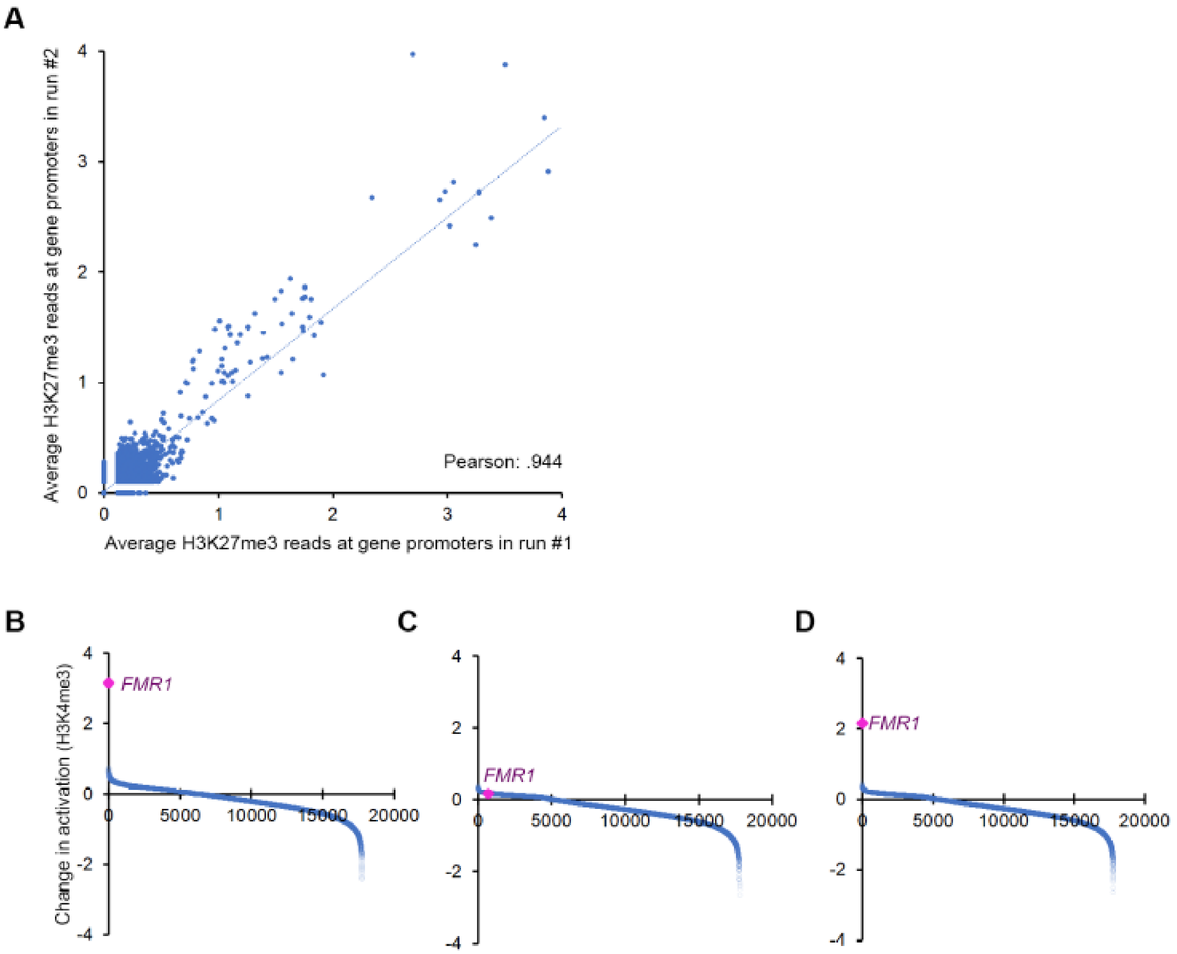

Supplemental Figure S6. CUT&RUN runs are highly reproducible and indicate the specificity of 5-aza-dC and 2HE-5NMe for *FMR1*. (*A*) Replicate to replicate comparison of CUT&RUN data. Reproducibility of CUT&RUN runs is very high as measured by Pearson's correlation coefficient. Two independent replicates were conducted to determine genome-wide locations of H3K27me3 in untreated FXS neurons (WCMC37). Linear regression of the data is indicated by the dotted line. (*B*) Gene ranking of changes in activating histone marks indicates that 5-aza-dC induces a specific change in H3K4me3 occupancy at the *FMR1* promoter. Shown is a scatterplot graphing the change in H3K4me3 occupancy against the ranking of all genome promoters by the numerical change in H3K4me3 occupancy. (*C*) Withdrawal of 5-aza-dC and culturing in DMSO leads to drop in H3K4me3 occupancy and gene ranking. Scatterplot prepared as in S3B. (*D*) Withdrawal of 5-aza-dC and culturing in 2HE-5NMe maintains H3K4me3 marks at the *FMR1* promoter and gene ranking. Scatterplot prepared as in S3B.

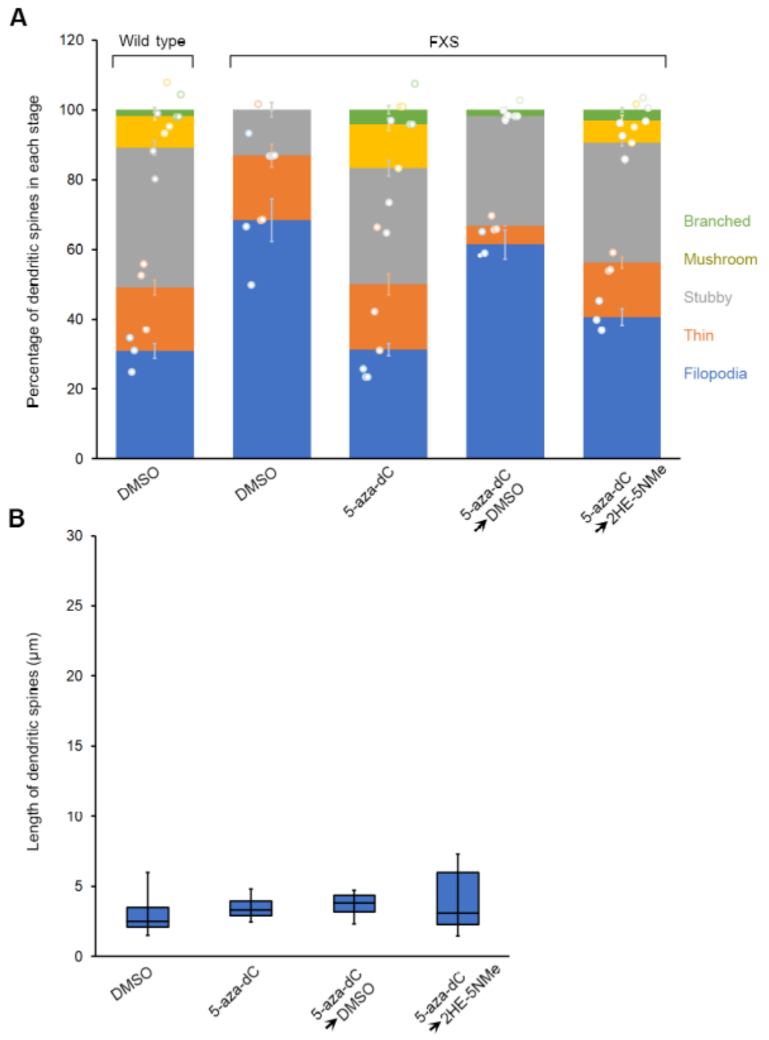

Supplemental Figure S7. Defective FXS dendritic spine maturation can be restored with drug treatments. (*A*) Defective dendritic spine maturation in FXS neurons (WCMC37) can be restored with 5-aza-dC and maintained with 2HE-5NMe. Quantification of each class of dendritic spine in wild-type neurons or FXS neurons following treatments (n = 3 experiments, mean and s.d.; univariate two-sided t-test). (*B*) The spine length of dendritic spines in wild-type neurons (WCMC7) is unaffected when the neurons are treated with 5-aza-dC which indicates an FXS neuron-specific effect. Quantification of dendritic spine length (µm) in wild-type neurons following treatments (Box limits, interquartile range; whiskers, minimum to maximum; center line, median; univariate two-sided t-test).
